# Primary Squamous Cell Carcinoma of Lung Leading to Metastatic Jaw Tumor

**DOI:** 10.1155/2014/392616

**Published:** 2014-11-10

**Authors:** Chintamaneni Raja Lakshmi, M. Sudhakara Rao, Sujana Mulk Bhavana, Sivan Sathish

**Affiliations:** ^1^Department of Oral Medicine and Radiology, Drs Sudha and Nageswara Rao Siddhartha Institute of Dental Sciences, Gannavaram Mandal, Krishna District, Andhra Pradesh 521286, India; ^2^Department of Otorhinolaryngology, Dr. Pinnamaneni Siddhartha Institute of Medical Sciences and Research Foundation, Gannavaram Mandal, Krishna District, Andhra Pradesh 521286, India; ^3^Department of Oral Medicine and Radiology, Chettinad Dental College & Research Institute, Rajiv Gandhi Salai, Kelambakkam, Kancheepuram District 603103, India

## Abstract

Metastatic tumors to the orofacial region are unusual and they may occur in the oral soft tissues or jaw bones. Owing to their clinical variability the diagnosis of such tumors is often a dilemma. We report a unique case of mandibular metastasis which became the first evidence of an occult primary in the lung.

## 1. Introduction

Metastatic tumors to the oral cavity are uncommon comprising only 1–3% of all malignant oral neoplasms. They usually involve oral soft tissues and jaw bones [1]. According to the literature, metastatic tumors in the oral region primarily originate from the breast, followed by lung, kidney, thyroid gland, intestine, prostate gland, stomach, testis, and bladder. Mandible is the most common site for metastases, predominantly occupying molar region [2]. Oral metastasis pose a diagnostic challenge to the dental practitioner as their clinical findings often mimic reactive or benign lesions and even odontogenic infections. These metastatic tumors have great clinical significance as they may be the first evidence of an undiscovered malignancy at a distant primary site. The aim of this paper is to illustrate a rare case of squamous cell carcinoma of lung with metastases to the mandible causing severe trismus as the initial manifestation of disease.

## 2. Case Report

**Figure 1:**
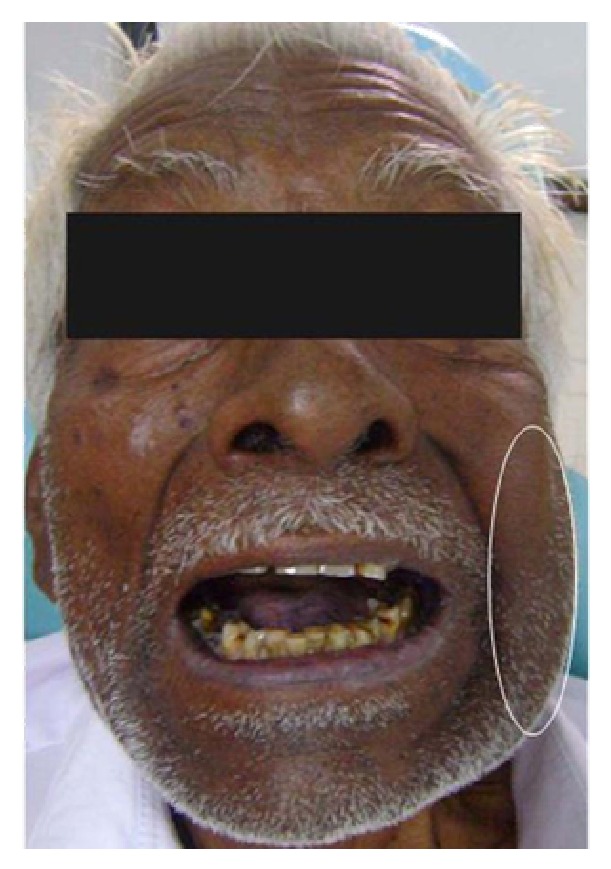
Swelling on left side of the face with associated trismus.

A 78-year-old male patient reported with a complaint of swelling on left side of the face (Figure 1) and difficulty in mouth opening since 4 months with associated paresthesia of the same region for the past 2 months.

**Figure 2:**
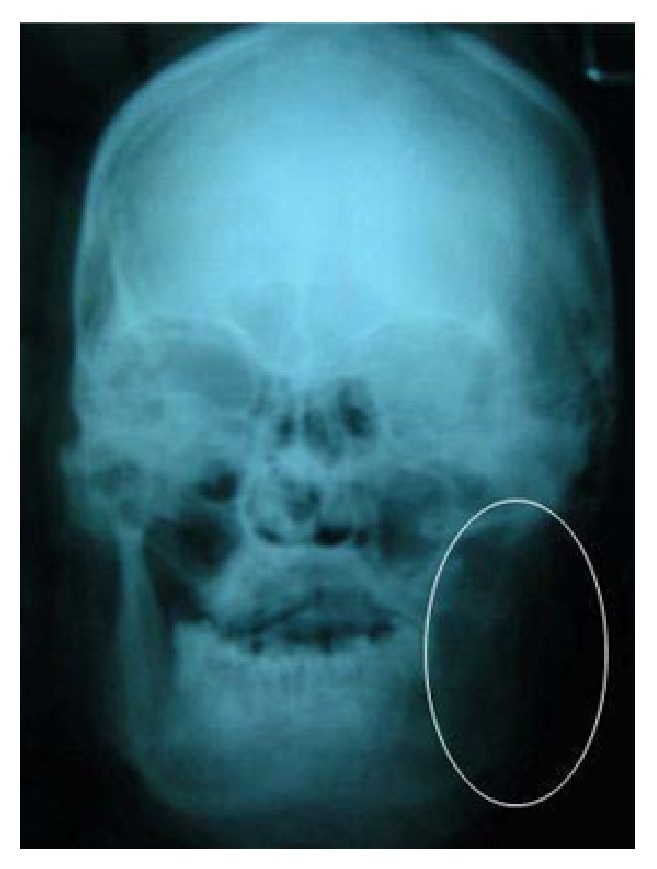
Posteroanterior view of skull revealing osteolysis involving left body and ramus of the mandible till the condylar head.

He revealed productive cough and exertional dyspnoea for the past 4 years and was under steroid inhalers. Patient had habit of smoking 5-6 chuttas per day for the past 50 years. Clinical examination revealed diffuse swelling over left body and angle of the mandible. Skin over the swelling was smooth with no surface changes. On palpation it was firm in consistency and slightly tender. There was also a palpable lymph node in the left sub mandibular region which was 2 × 1.5 cm in size, hard in consistency, nontender, and not freely movable. Intraoral examination revealed decreased mouth opening with inter incisal distance of 24 mm, restricted jaw movements, and grade 2 mobility in relation to 36, 37, and 38. Considering history and clinical picture provisional diagnosis of malignant neoplasm involving left body and angle of mandible was considered. Differential diagnosis of metastatic jaw tumor, osteosarcoma, and chondrosarcoma was given. Panoramic and PA skull radiographs revealed radiolucency with irregular borders at the left body and ramus of the mandible till the condylar head (Figure 2).

**Figure 3:**
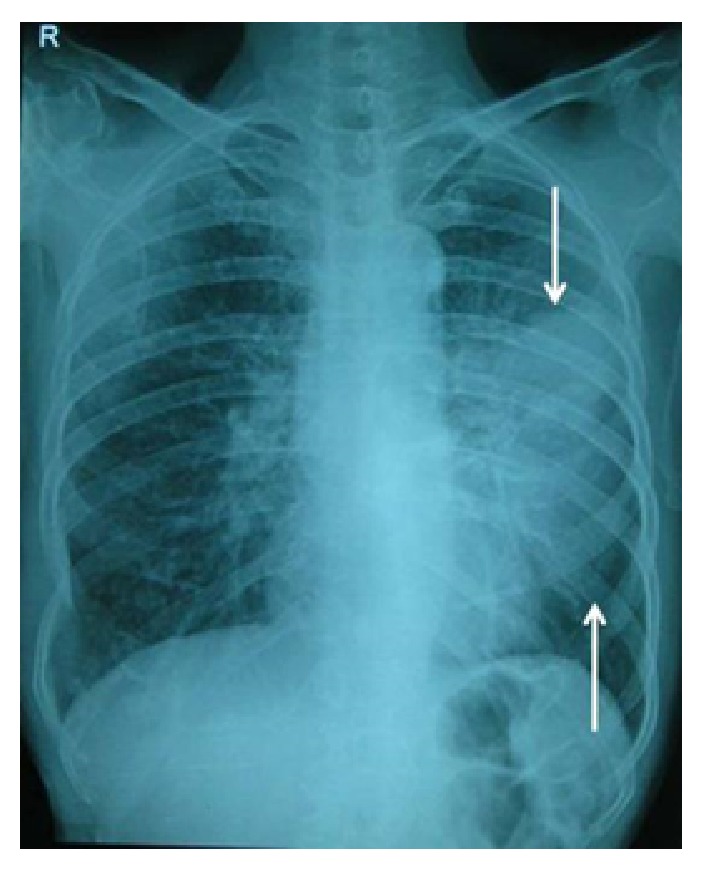
Chest radiograph revealed a homogeneous opacity involving the entire mid- and lower zones of the left lung.

TMJ tomography showed osseous destruction involving left condyle. Radiographically differential diagnosis of gorhams disease and metastatic jaw tumor were considered. Considering history of persistent cough chest radiograph was taken which revealed homogeneous opacity involving the entire mid- and lower zones of the left lung (Figure 3).

**Figure 4:**
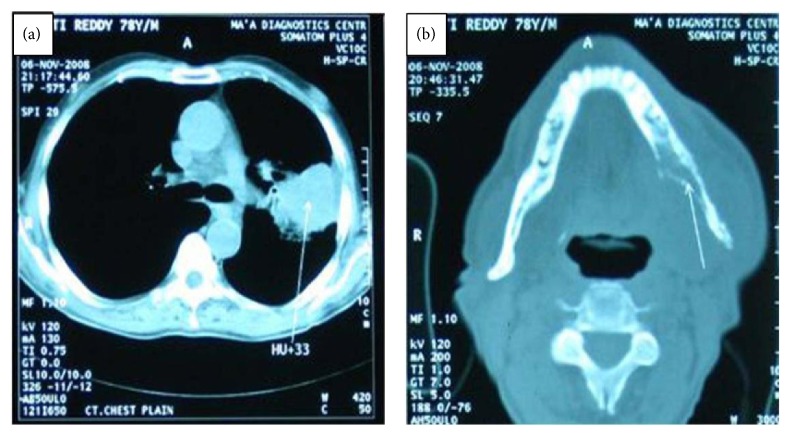
(a) CT chest revealed lesion in the left lung. (b) Axial sectional study of CT mandible revealed osteolysis of left body and ramus of the mandible with adjacent soft tissue mass.

The patient was referred to radiologist for CT chest and mandible. CT chest revealed 12 × 7 × 6 cm mixed dense lesion in left lung with bronchial stenosis and distal collapse (Figure 4(a)). Axial sectional study of CT mandible revealed osteolytic areas involving the left body extending from 46 region of mandible encroaching the entire ramus till the condylar head with adjacent soft tissue mass (Figure 4(b)).

**Figure 5:**
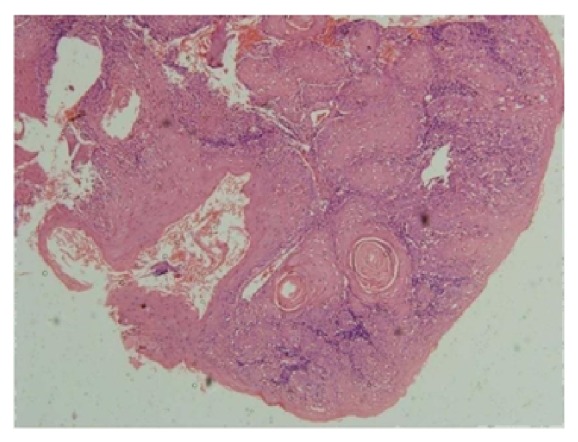
Histopathological picture revealing features suggestive of squamous cell carcinoma.

As metastatic work up abdominal ultrasound scan was done which revealed no evident pathology. Later whole body scan was performed showing metastasis in the mandible and left submandibular lymph node. Hematological findings showed raised ESR. Serum calcium, phosphorus, alkaline, and acid phosphatase were done to rule out fibro osseous lesions which were under normal limits. Incisional biopsy was done from jaw tumor which revealed infiltrating epithelial islands with dysplastic features like cellular pleomorphism, nuclear hyperchromatism, increased nuclear cytoplasmic ratio, mitotic figures, keratin pearls, and dense inflammatory cell infiltrate suggestive of squamous cell carcinoma (Figure 5).

**Figure 6:**
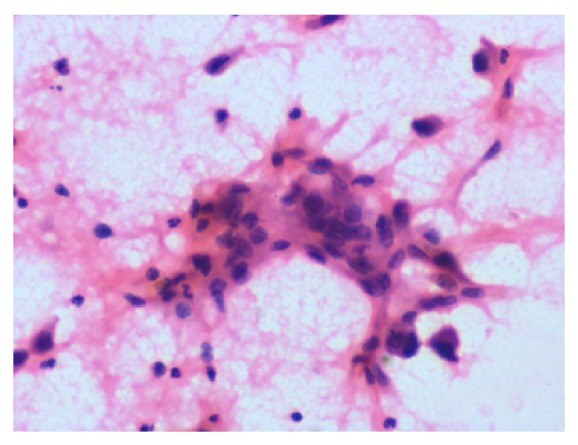
Cytological smear from lung tumor showing dysplastic cells in inflammatory background suggestive of squamous cell carcinoma.

**Figure 7:**
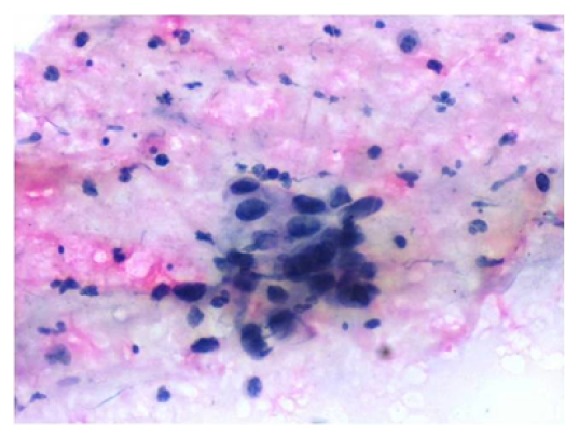
Cytological smear from left submandibular lymph node showing squamous dysplastic islands suggestive of squamous cell carcinoma.

Cytological smear from lung tumor showed few discrete atypical epithelial cells with eosinophilic cytoplasm and irregular hyperchromatic nuclei with evident keratinisation suggestive of squamous cell carcinoma (Figure 6). FNAC was performed from left submandibular lymph node which revealed squamous dysplastic islands suggestive of squamous cell carcinoma (Figure 7).

Considering clinicoradiographic features and histopathological findings final diagnosis of mandibular metastasis with primary squamous cell carcinoma of lung was given and patient was referred to oncologist for further evaluation. Sadly patient expired within 4 months after discovery of metastatic lesion in the mandible. Consent was taken from patient's son regarding publication.

## 3. Discussion

Lung cancer has increased in incidence throughout the twentieth century and is now the most common cancer in the world. It has a poor prognosis, only 10–15% of patients survive 5 years or longer. There is a strong correlation between tobacco chewing, smoking, and the development of lung cancer. Oral metastasis is considered as a late complication and frequently associated with multiple organ metastases. Lung is the most common source for cancers that metastasize to the oral soft tissues and breast is the most common primary site for tumors that metastasize to the jawbones [3]. Current case is atypical with lung malignancy metastasizing to jaw bone. When a meta-analysis was done from 1995 to 2011 by Shin et al., out of 1445 oral malignancies, twenty nine cases of metastasis were retrieved comprising only 2% [4]. In a study Daley and Darling identified 7 cases out of 38 metastatic tumors, as having primary site in lung with metastasis involving different sites of oral cavity [5]. Most metastatic tumors to the orofacial region are commonly seen in patients between 40 and 70 years with gender distribution of male to female ratio 2 : 1 [6]. In the present case patient was a 78-year-old male. Pathogenesis of metastasis to jaw bones is unclear but possible predilection for mandible is due to large amount of red bone marrow and increased flow of the circulating blood. Metastasis to the jaw bones may produce a variety of signs and symptoms including swelling, pain, loose teeth, paresthesia, and trismus. Symptoms like numb chin or mental nerve neuropathy should always predict metastatic disease of the bone involving inferior dental or mental nerves. These features when seen in patients with known malignancy are termed as “mental nerve neuropathy” or the “numb chin syndrome” [7]. Our patient had symptoms like loose teeth, swelling, pain, paresthesia, and trismus supporting the diagnosis of metastatic jaw tumor.

Bone metastasis shows two main radiographic appearances:

1. Frank destruction of bone with new bone formation within the lesion or adjacent bone;
2. appearance mimicking osteomyelitis characterized by presence of many areas of destruction. Most of the bony metastatic lesions from kidney, lung, or breast cancers are more often osteolytic, whereas metastases from prostate cancer often form osteoblastic lesions in bone. Mandibular condyle and TMJ metastasis of lung squamous carcinoma is extremely rare [8]. The present case depicted osteolytic areas involving left body, ramus of mandible, and left condylar head.

Correct diagnosis is further complicated because such osteolytic metastatic lesions of the mandible can mimic bone infections, granuloma of the bone, benign tumors, primary tumors with or without extension to the adjacent tissue, systemic disease involving the jaw bone, for example, Histiocytosis X, and jaw bone involvement in systemic malignancy, for example, Multiple myeloma, fibro-osseous lesion, and giant cell lesion. The irregular border of osteolytic lesion may give some clue to the metastatic lesion which is evident in the present case. Temporomandibular joint metastasis should also be considered as differential diagnosis in patients presenting with trismus [9].

Huang et al. described acceptable criteria for metastatic tumors to the jaw bones as follows: [9]

1. a provided primary tumor with histopathologic confirmation and roentgenographic evidence;
2. maxillary, mandibular, or mucosal metastasis with histopathologic confirmation and roentgenographic evidence;
3. histopathologic correlation of metastatic lesion;
4. in the event of primary lesion anatomically near the metastasis, direct extension must be ruled out by a wide, clear margin around the primary site, with no tumor tissue present between the two foci.

In the present illustrated case histopathological correlation of primary and metastatic tumor was done which revealed squamous cell carcinoma of both lung and jaw tumor, along with roentgenographic evidence.

Metastatic tumors in the jaw bones are difficult to recognize for a number of reasons, such as

1. the lesions are centrally located in the bone,
2. very few subjective symptoms except at a very late stage,
3. radiographs which are usually nonspecific [5, 6].

The treatment option and prognosis depends upon site of origin and degree of metastatic spread. In a study conducted by Adebayo and Ajike, 19 of 24 cases (79%) of oral metastatic tumors died within 12 months of diagnosis which attributed to the poor prognosis of oral metastasis [10]. Treatment modalities include surgical resection, radiation, chemotherapy, or a combination of these techniques. In case of recurrent primary tumors, elderly individuals with underlying systemic diseases can be managed conservatively with palliative therapy to avoid functional disabilities. Unfortunately, the discovery of oral metastatic tumor usually represents a terminal disease and poor overall prognosis [7, 10]. In the present case mandibular metastasis was the first sign of the underlying squamous cell carcinoma of lung. Patient was explained about the treatment options and was referred to the oncology centre for further management. Sorrowfully patient expired within a period of 4 months after the diagnosis of oral metastasis.

## 4. Conclusion

In conclusion, the present case highlights a very unusual incidence of mandibular metastasis from squamous cell carcinoma of lung. Careful examination and clinical suspicion is mandatory as they mimic odontogenic infections and benign tumors. Early diagnosis of such lesions is crucial as they limit treatment options in late stages resulting in grave prognosis.
